# Supplementary material for: High resolution mapping of the tumor microenvironment using integrated single-cell, spatial and in situ analysis
Source: Nat Commun. 2023 Dec 19;14:8353. doi: 10.1038/s41467-023-43458-x (PMC10730913; doi:10.1038/s41467-023-43458-x)
Supplement: Supplementary file 1 — Supplementary Information [file 41467_2023_43458_MOESM1_ESM.pdf]

Supplemental Information

Supplemental Figures

Supplemental Figure 1

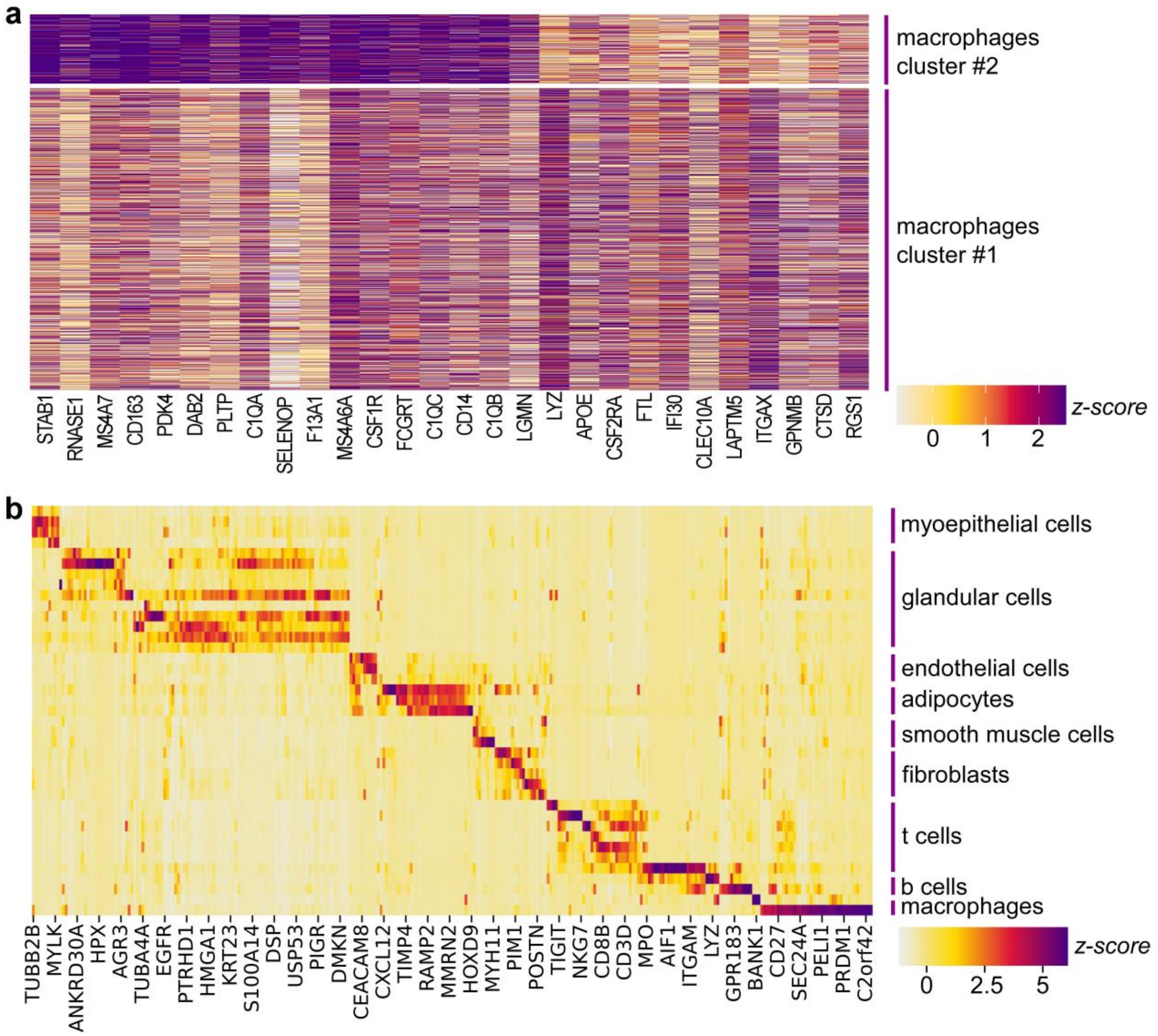

Supplemental Figure 1. a Differentially expressed genes between the two macrophage clusters identified in the scFFPE-seq data. Z-score computed across cell types for each gene.
b Xenium Gene Panel Design. The 280 gene pre-designed Xenium human breast panel was combined with 33 add-on genes (Supplemental Table 2), selected based on single cell data of human breast tissue[1–3] and manual curation. The heatmap shows the relative expression of genes across different cell types found in the references used to build the panel. The gene markers chosen are generally mutually exclusive for their cell type. Z-score computed across cell types for each gene.

Supplemental Figure 2

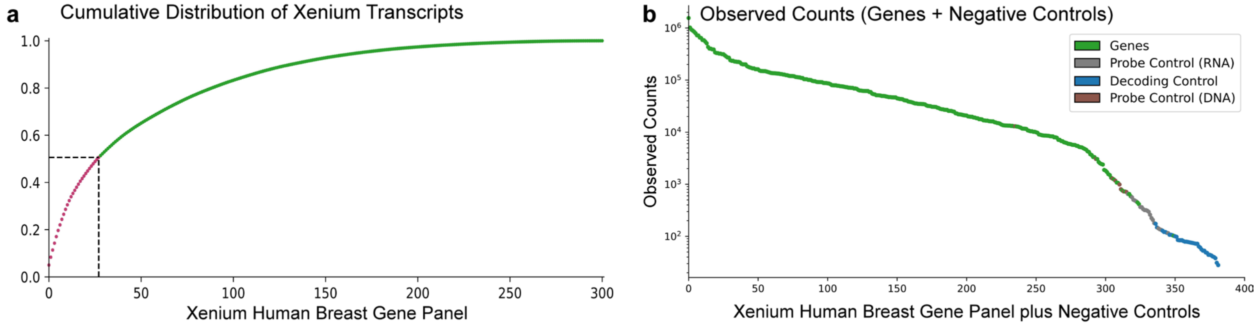

Supplemental Figure 2. Xenium Quality Control Metrics. a Complexity measurement showing the cumulative distribution plot of total transcripts contributing to genes on the Xenium Human Breast Panel. Dotted line at y = 0.5 signifies that 50% of the total transcripts observed contribute to 27 genes. **b** Knee plot showing observed counts (Q ≥ 20) of genes and negative controls: 1) probe controls to assess non-specific binding to RNA, 2) decoding controls to assess misassigned genes, and 3) genomic DNA (gDNA) controls to ensure the signal is from RNA.

Supplemental Figure 3

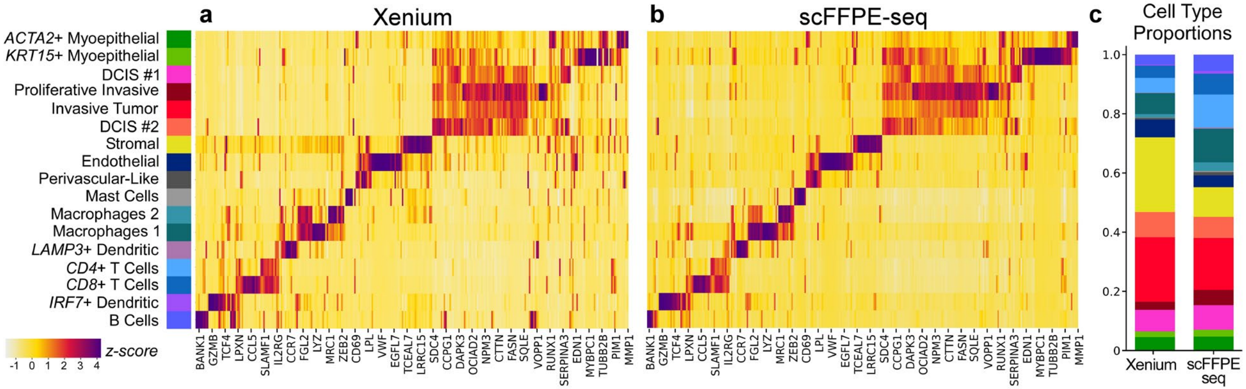

Supplemental Figure 3. Relative expression of genes across cell types comparing Xenium and scFFPE-seq. a, b Heatmap representation of the t-SNEs in Fig. 3i (scFFPE-seq, down-selected to 313 genes) and Fig. 3j (Xenium), demonstrating that the cell type representation is generally similar. Z-score computed across cell types for each gene, by subtracting the mean and dividing by the standard deviation. **c** Comparison of cell type proportions in Xenium versus scFFPE-seq.

Supplemental Figure 4

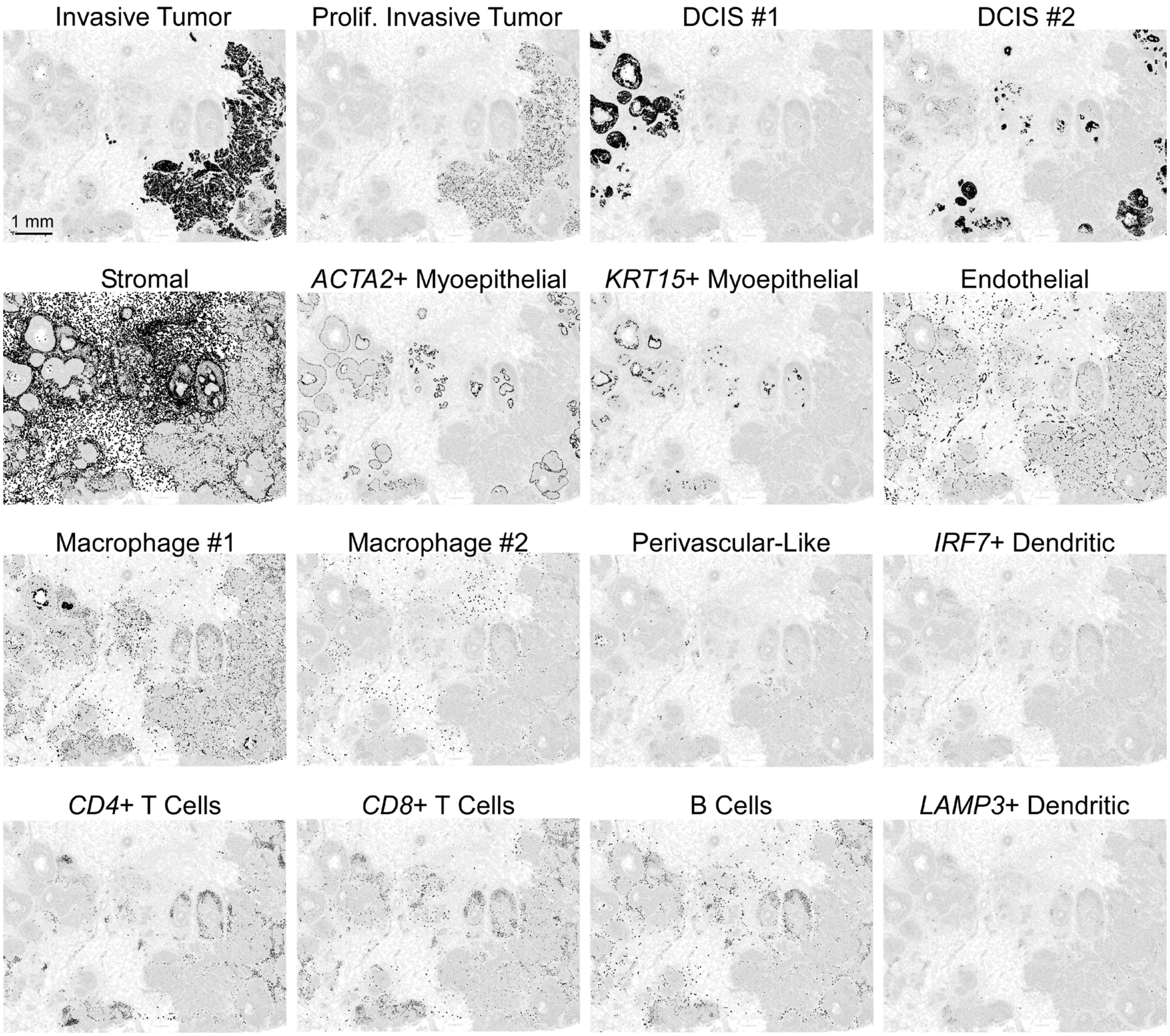

Supplemental Figure 4. All cell types pictured in Fig. 3l are shown here individually. The highlighted cell type is filled black, and all other cells are filled white. Scale bar = 1 mm. This experiment was performed in replicate on two serial sections, with one representative section shown here.

Supplemental Figure 5

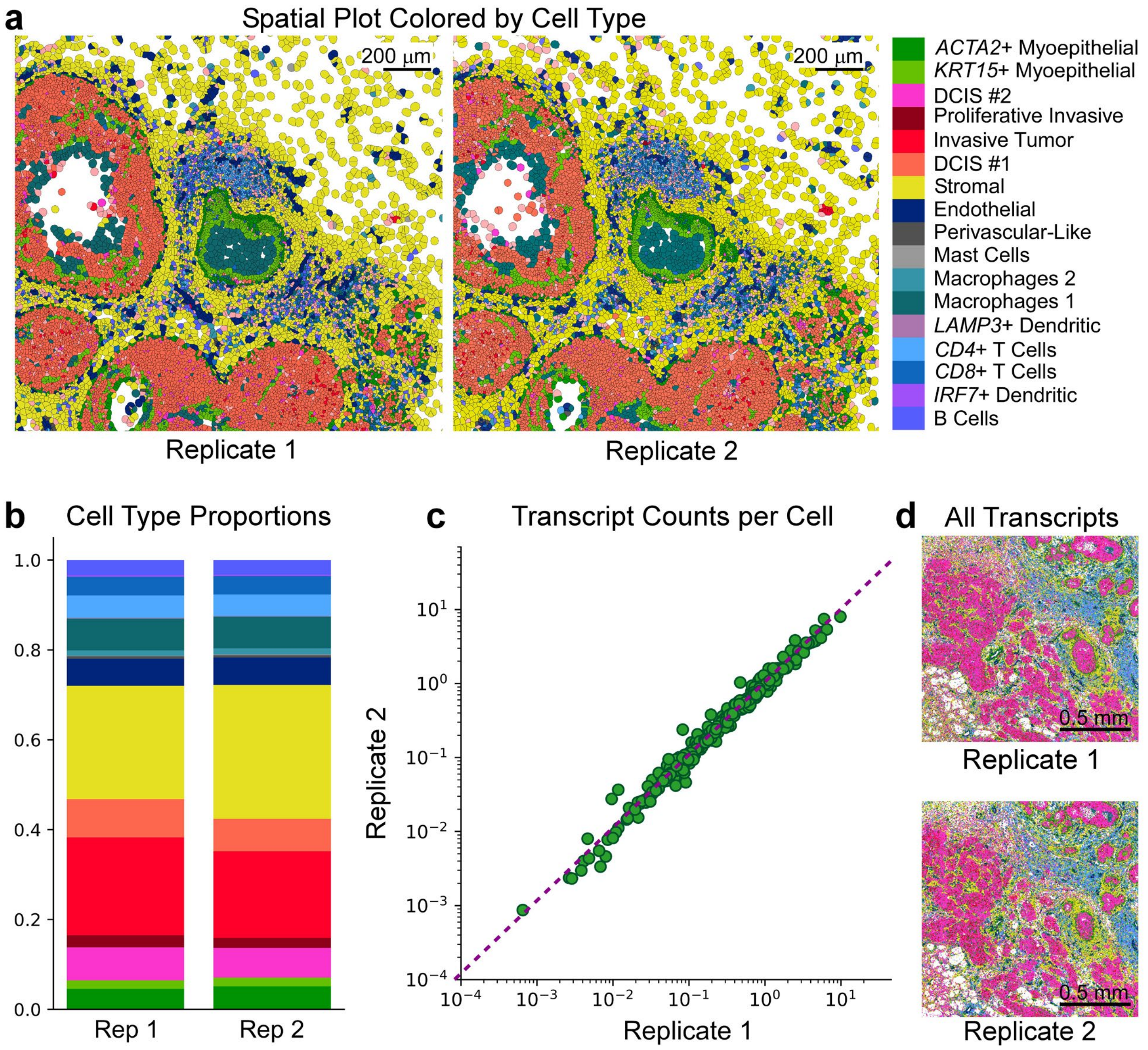

Supplemental Figure 5. Serial section Xenium replicates are highly correlated. a Cell types for each replicate from the same ROI. Scale bar = 200 µm. **b** Cell type proportions across the whole section. **c** Scatter plot comparing transcripts per cell for each gene across replicates (r^2 = 0.99). **d** ROI showing all transcripts, color coded by cell type, for each replicate. Scale bar = 500 µm.

Supplemental Figure 6

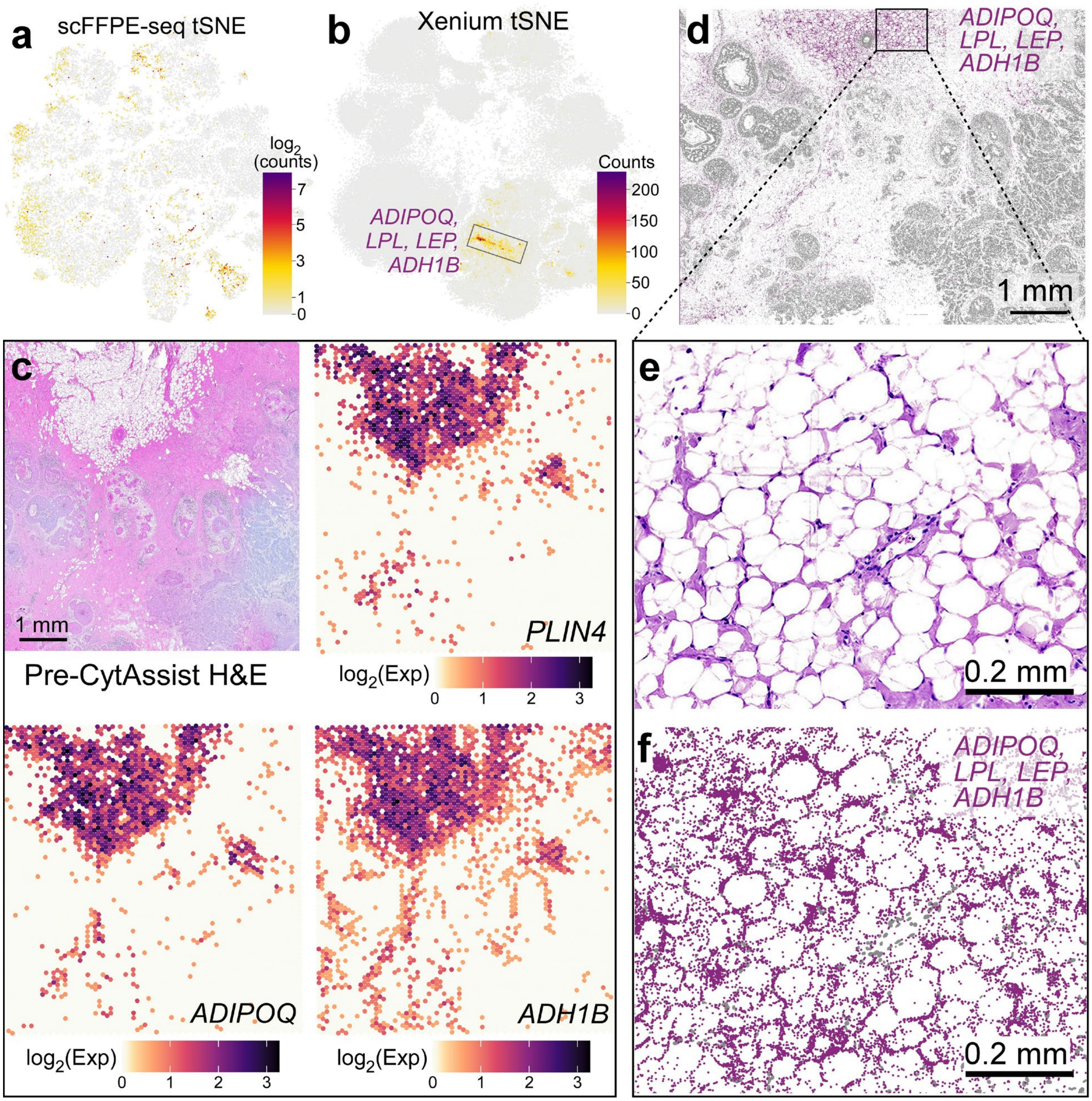

Supplemental Figure 6. Visium and Xenium data identify adipocytes and their gene markers. a t-SNE projection of the scFFPE-seq data showing the combined expression of 11 adipocyte markers (*FABP4*, *GPX3*, *PLIN1*, *PLIN4*, *ADH1B*, *ADIPOQ*, *G0S2*, *LPL*, *GPD1*, *LIPE*, *SLC19A3*). See Fig. 2a for cluster annotations. **b** t-SNE projection of the Xenium data displaying the expression of all adipocyte markers on the Human Breast Panel: *ADIPOQ*, *LPL*, *LEP*, and *ADH1B*. **c** H&E staining conducted pre-CytAssist is shown for reference alongside the Visium spatial distribution of three known adipocyte markers (*PLIN4*, *ADIPOQ*, and *ADH1B*) expressed as $\log_2$(normalized UMI counts). Scale bar = 1 mm. **d** Spatial plot of the Xenium data displaying the expression of all adipocyte markers on the Human Breast Panel: *ADIPOQ*, *LPL*, *LEP*, and *ADH1B*. Scale bar = 1 mm. **e**, **f** Closer view of an adipocyte region showing **e** post-Xenium H&E and **f** Xenium spatial plot for adipocyte markers with nuclei in gray. Scale bar = 0.2 mm. Both the Xenium and Visium experiments were performed in replicate on two serial sections, with one representative section from each technology shown here.

Supplemental Figure 7

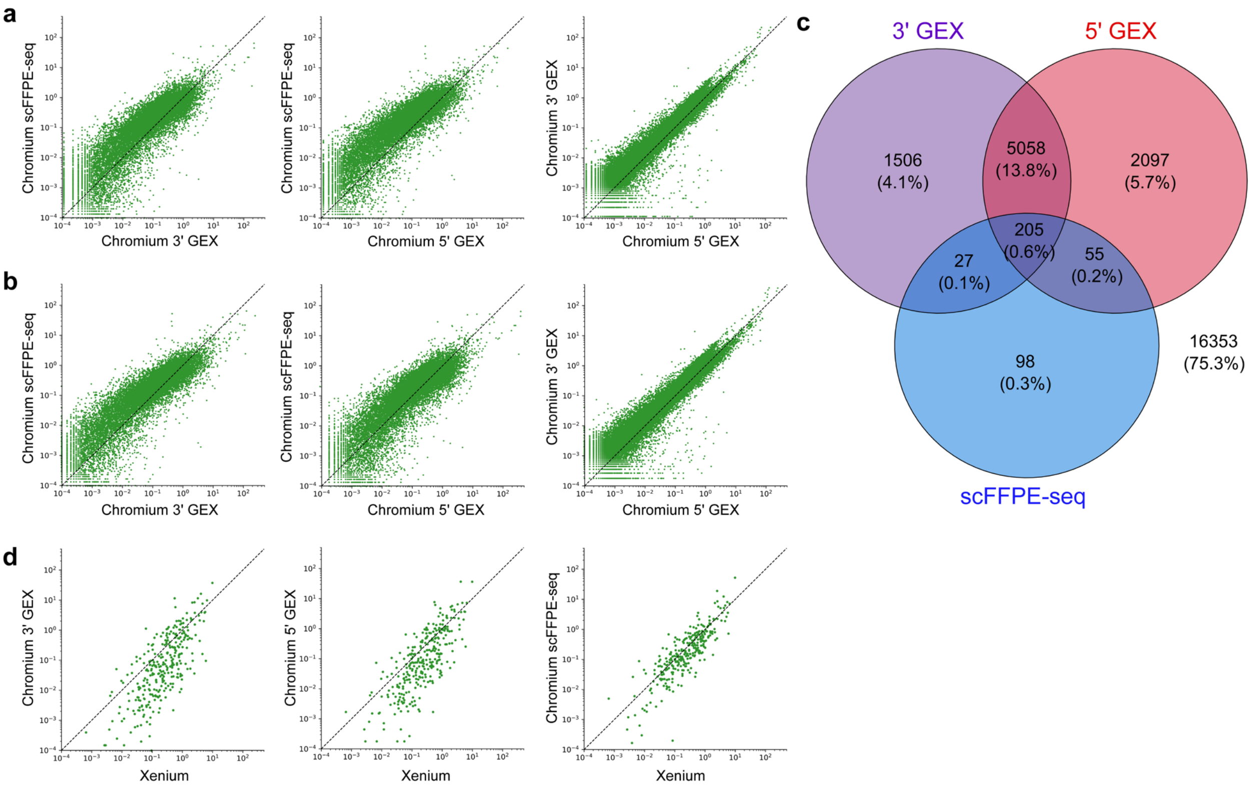

Supplemental Figure 7. Benchmarking scFFPE-seq and Xenium sensitivity from FFPE curls against Chromium 3′ and 5′ GEX from patient-matched FF dissociated tumor cells. a Scatter plots of UMIs per cell representing pairwise comparisons of Chromium single cell technologies (scFFPE-seq, 3′ GEX and 5′ GEX) at the recommended sequencing depth for scFFPE-seq (10,000 mean reads per cell). **b** Scatter plots of UMIs per cell in pairwise comparisons between scFFPE-seq, 3′ GEX and 5′ GEX at the recommended sequencing depth for 3′ GEX and 5′ GEX (20,000 mean reads per cell). **c** Venn diagram of the overlap between genes with zero counts across all cells within each of the three sequencing platforms at 10,000 mean reads per cell. The number “16353” outside of the Venn diagram represents the genes which are expressed with greater than zero counts and are possible to detect in all technologies **d** Scatter plots of UMIs per cell and transcripts per cell between Chromium at the recommended sequencing depth and Xenium. Genes are downsampled to those contained within the Xenium panel. Dotted lines represent X=Y.

Supplemental Figure 8

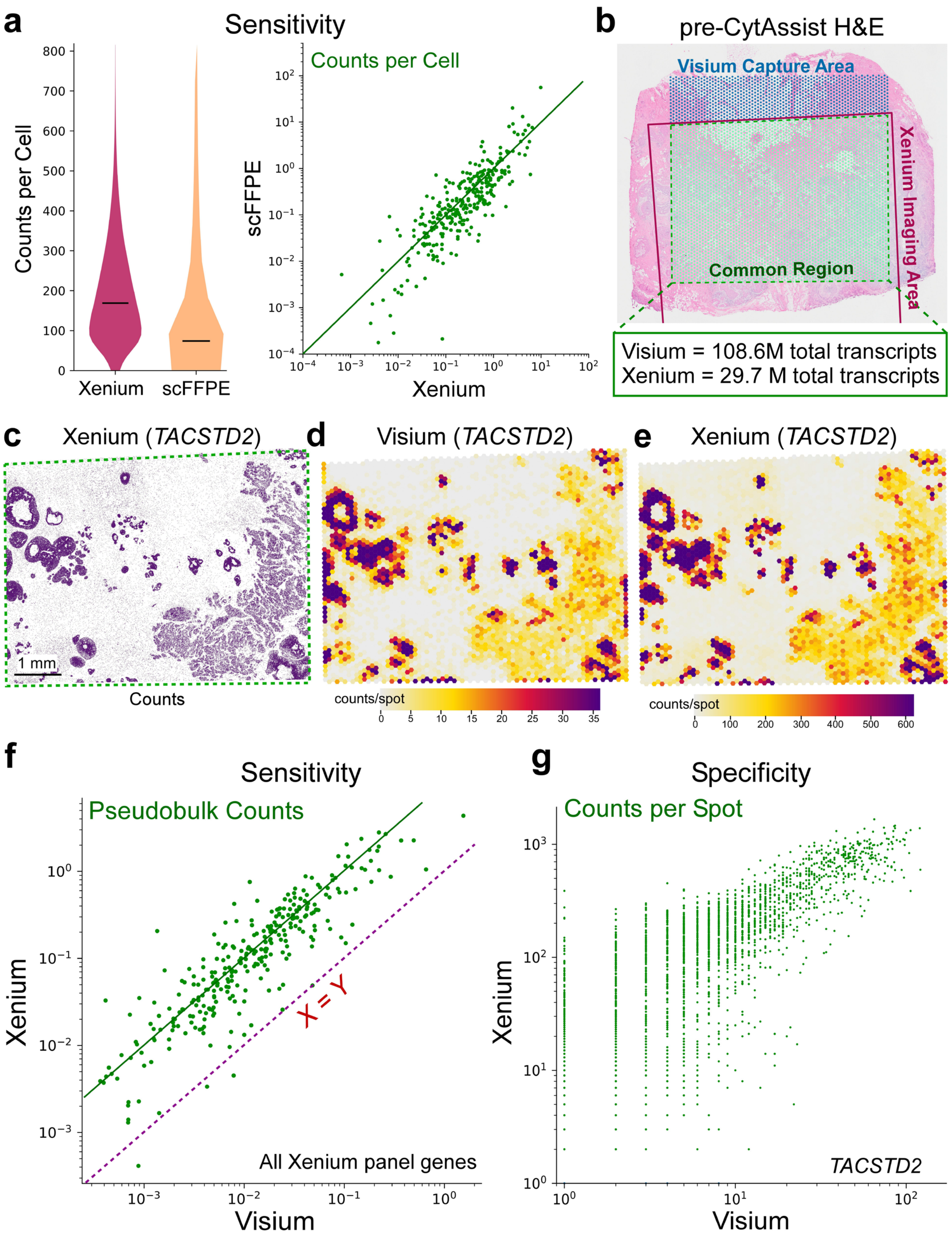

Supplemental Figure 8. Xenium, Visium and scFFPE-seq are benchmarked for specificity, sensitivity, and resolution. a Chromium scFFPE-seq data down-selected for only the 313 genes that appear on the Xenium gene panel. Violin and scatter plots showing the total number of transcript counts per cell detected in the Xenium data vs. UMIs per cell in the scFFPE-seq data. Pearson's R^2=0.66; Spearman's r^2 = 0.85 (using log mean counts per cell). **b** Registration of Visium and Xenium H&E allows for the isolation of a common area between the two platforms. The number of total transcripts within this common area is reported. **c-e** Tumor epithelial marker *TACSTD2* gene expression is shown as **c** Xenium transcript localization plot (decoded at Q ≥ 20), **d** Visium spatial plot (counts/spot), and **e** pseudobulk Xenium expression counts mapped to Visium spots. Resolution improvements from Visium are notable in the Xenium data, even at low magnification (compare **c** and **d**). Scale bar = 1 mm. **f** Sensitivity scatter plot expressed as pseudobulk counts per spot, quantified for all 313 genes on the Xenium panel. Dotted line represents X=Y; solid line is 10X = Y. **g** Comparison of Xenium and Visium data (counts per spot) showing high spatial correspondence (r^2 = 0.88).

Supplemental Figure 9

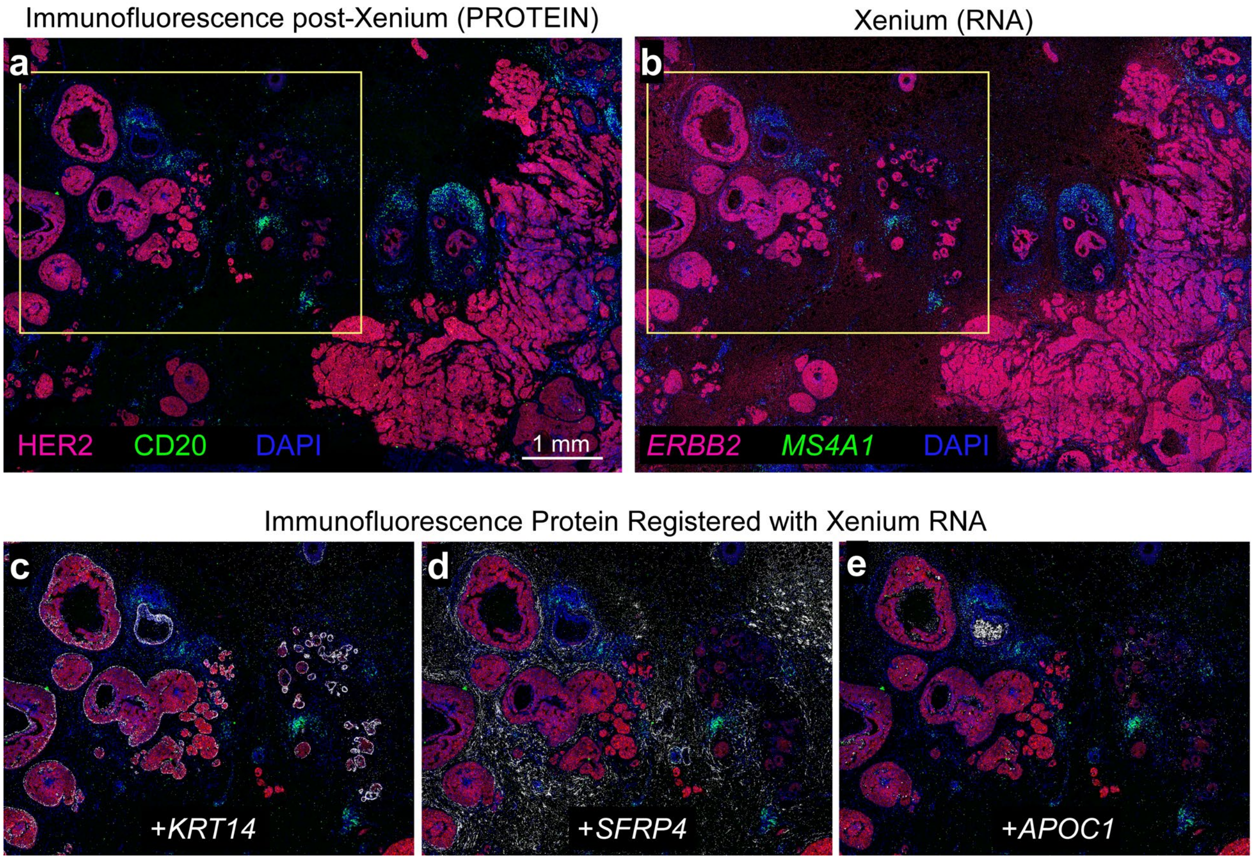

Supplemental Figure 9. Protein immunofluorescence images acquired post-Xenium from the same section validate the specificity of Xenium RNA probes. a After 15 cycles of imaging on the Xenium instrument, sections were immunostained for HER2 and CD20 proteins using fluorescent secondary antibody detection in the 594 and 488 channels, respectively. Scale bar = 1 mm. **b** Comparing the equivalent Xenium RNAs (*ERBB2* and *MS4A1*), spatial correlation to protein expression was nearly identical. **c-e** Region of interest outlined with yellow box in **a** and **b**. Because RNA and protein data were obtained from the same section, the two DAPI images were registered and overlaid with RNA and protein expression. HER2 and CD20 protein immunofluorescence are shown registered with **c** *KRT14* RNA (myoepithelial), **d** *SFRP4* RNA (stromal), and **e** *APOC1* RNA (macrophages). Immunofluorescence was performed on both serial sections post-Xenium with one representative section shown here.

Supplemental Figure 10

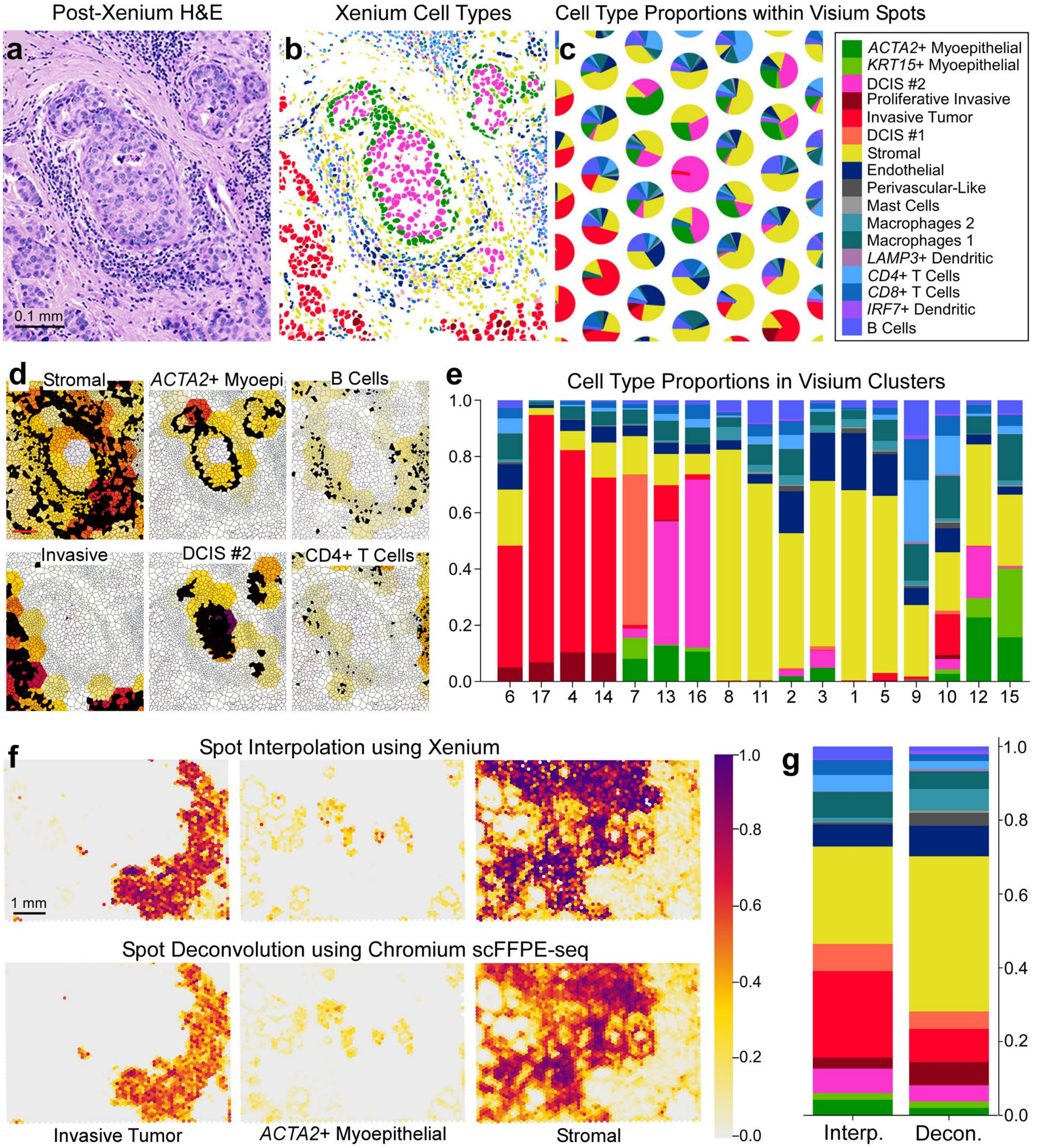

Supplemental Figure 10. Xenium and scFFPE-seq assign cell type frequencies to individual Visium spots. a-c Registration of Visium and Xenium H&E images (see Supp. Fig. 8b) allows for the interpolation of cell type proportions within Visium spots using Xenium transcript counts. Scalebar = 0.1 mm. **d** Annotated cell types (see Supp. Fig. 4) from Xenium are overlaid on a Visium heatmap expressed as a proportion (fraction of one) of that cell type within each spot (see scale bar in **f**). Scale bar = 0.1 mm. **e** Interpolation of Visium spots allows for mixed, unannotated cell clusters to be resolved into cell type frequencies. **f** We deconvolved Visium spots into cell types with spacexr[4] using the scFFPE-seq data as the single cell reference. Scale bar = 1 mm. **f, g** Comparison of the spot interpolation method using Xenium and the deconvolution method using scFFPE-seq. Cell type proportions are expressed as a fraction of one.

Supplemental Figure 11

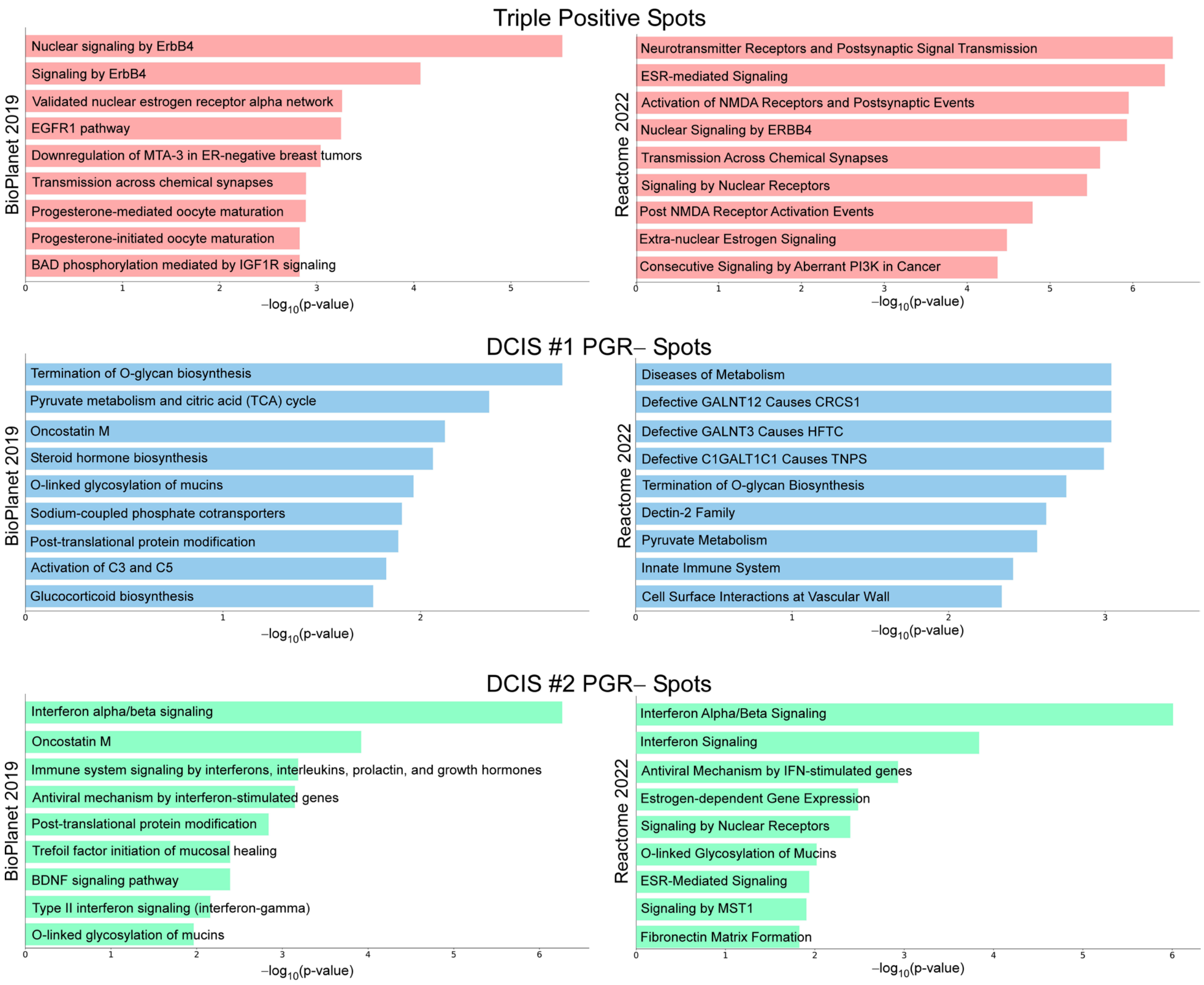

Supplemental Figure 11. Enriched gene ontology terms from two different databases (BioPlanet 2019 and Reactome 2022) associated with triple positive, DCIS #1 (PGR−), DCIS #2 (PGR−) Visium spots.

Supplemental Figure 12

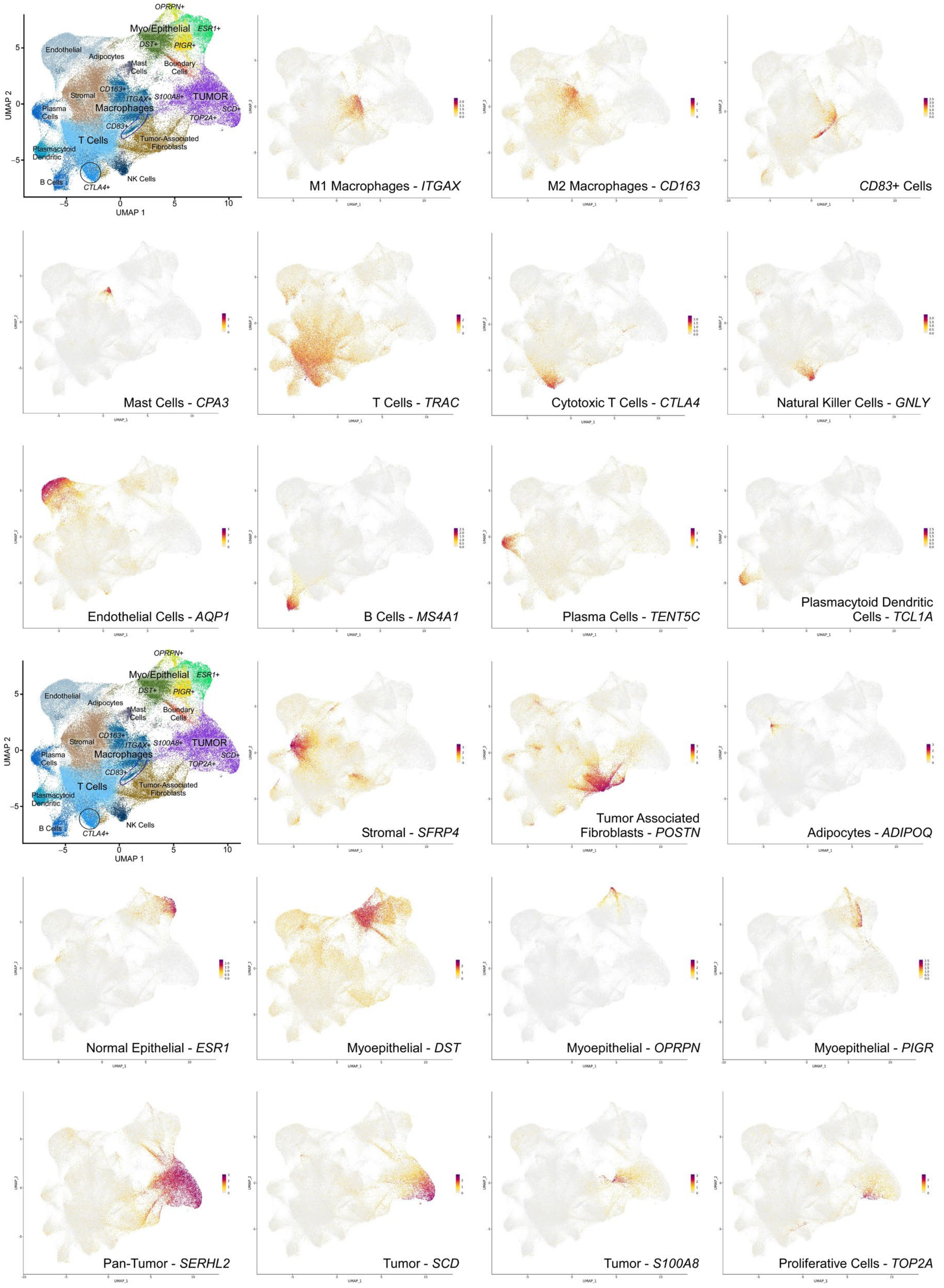

Supplemental Figure 12. Individual cell types in Figure 6 can be identified with a single marker. t-SNE projection of the Xenium data and selected gene markers.

Supplemental Figure 13

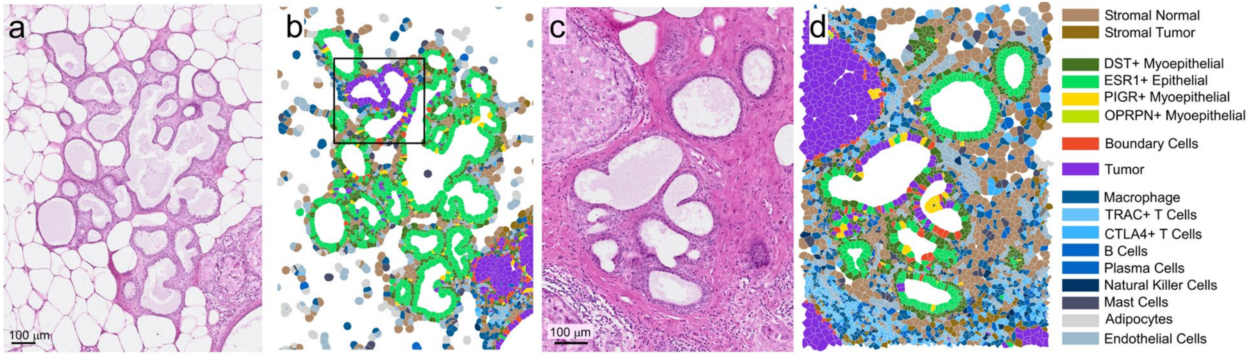

Supplemental Figure 13. Normal-appearing ducts contain tumor cells. a H&E of one FOV of a normal duct region amidst adipocytes. Scale bar = 100 µm. **b** Cells are labeled according to the clusters provided in Fig. 6a. Boxed region shows tumor cells present where duct morphology appears normal. **c** H&E of a different FOV of a normal duct region. Scale bar = 100 µm. **d** Cells are labeled according to the clusters provided in Fig. 6a. Tumor cells are again present where duct morphology appears normal.

Supplemental Information

Supplemental Table 1. Metrics

Flex Sample #1	Tissue (Curl) Size	Tissue Type and Thickness	Number of Cells	Total Transcripts	Median Reads per Cell	Median Genes per Cell	Genes Targeted	Genes Detected	Median UMI Counts per Cell	Reads Mapped Confidently to Probe Set	Pipeline Version	Fraction mito-chondrial UMI counts	Estimated UMIs from Genomic DNA	Probe Set Name
	7.5 mm x 6 mm	50 µm FFPE curls	30,365	204,470,550	10,450	1,480	18,085	17,696	2,191	93.20%	celllranger-7.0.1	4.00%	0.04%	Human Transcriptome Probe Set v2

Visium Sample #1	Tissue (Capture) Size	Tissue Type and Thickness	Number of Spots Under Tissue	Total Transcripts	Mean Reads per Spot	Median Genes per Spot	Genes Targeted	Genes Detected	Median UMI Counts per Spot	Reads Mapped Confidently to Probe Set	Pipeline Version	Fraction mito-chondrial UMI counts	Estimated UMIs from Genomic DNA	Probe Set Name
	6.5 x 6.5 mm	5 µm FFPE	4,992	114,568,641	40,003	5,712	18,085	18,056	13,973	98.50%	spacerang er-2.0.0	6.60%	3.00%	Human Transcriptome Probe Set v2

Xenium Sample #1	Tissue (Imaging) Size	Tissue Type and Thickness	Number of Cells	Transcripts Decoded (Q-score ≥ 20)	Median Reads per Cell	Median Genes per Cell	Genes Targeted	Genes + Add-On	Median Transcripts per Cell	Fraction Counts Neg. Control Probes	Pipeline Version	Fraction Counts Decoding Controls	Fraction Counts from Genomic DNA	Probe Set Name
	7.5 mm x 6 mm	5 µm FFPE	167,885	36,944,521	NA	62	313	280+33	166	0.026%	2022.1204.1	0.01%	0.00%	Human Breast Panel

Xenium Sample #2	Tissue (Imaging) Size	Tissue Type and Thickness	Number of Cells	Transcripts Decoded (Q-score ≥ 20)	Median Reads per Cell	Median Genes per Cell	Genes Targeted	Genes + Add-On	Median Transcripts per Cell	Fraction Counts Neg. Control Probes	Pipeline Version	Fraction Counts Decoding Controls	Fraction Counts from Genomic DNA	Probe Set Name
	7.2 mm x 5.3	5 µm FFPE	142,272	12,919,042	NA	38	280	280	63	0.00%	1.4.0.6	0.00%	0.00%	Human Breast Panel

Supplemental Table 2. Xenium Gene List

Gene Name	ADD-ON?	Gene Name	ADD-ON?	Gene Name	ADD-ON?	Gene Name	ADD-ON?	Gene Name	ADD-ON?	Gene Name	ADD-ON?	Gene Name	ADD-ON?	Gene Name	ADD-ON?
ABCC11	BASE	*CCL8*	BASE	*CRISPLD2*	BASE	*FBLIM1*	ADD-ON	*KLRB1*	BASE	*MUC6*	BASE	*RAMP2*	BASE	*TCEAL7*	BASE
ACTA2	BASE	*CCND1*	BASE	*CSF3*	ADD-ON	*FBLN1*	BASE	*KLRC1*	BASE	*MYBPC1*	BASE	*RAPGEF3*	BASE	*TCF15*	ADD-ON
ACTG2	BASE	*CCPG1*	BASE	*CTH*	BASE	*FCER1A*	BASE	*KLRD1*	BASE	*MYH11*	BASE	*REXO4*	ADD-ON	*TCF4*	BASE
ADAM9	BASE	*CCR7*	BASE	*CTLA4*	BASE	*FCER1G*	BASE	*KLRF1*	BASE	*MYLK*	BASE	*RHOH*	BASE	*TCF7*	BASE
ADGRE5	BASE	*CD14*	BASE	*CTSG*	BASE	*FCGR3A*	BASE	*KRT14*	BASE	*MYO5B*	BASE	*RORC*	BASE	*TCIM*	BASE
ADH1B	BASE	*CD163*	BASE	*CTTN*	BASE	*FGL2*	BASE	*KRT15*	ADD-ON	*MZB1*	BASE	*RTKN2*	BASE	*TCL1A*	BASE
ADIPOQ	BASE	*CD19*	BASE	*CX3CR1*	BASE	*FLNB*	BASE	*KRT16*	ADD-ON	*NARS*	BASE	*RUNX1*	BASE	*TENT5C*	BASE
AGR3	BASE	*CD1C*	ADD-ON	*CXCL12*	BASE	*FOXA1*	BASE	*KRT23*	BASE	*NCAM1*	BASE	*S100A14*	BASE	*TFAP2A*	BASE
AHSP	BASE	*CD247*	BASE	*CXCL16*	BASE	*FOXC2*	BASE	*KRT5*	BASE	*NDUFA4L2*	BASE	*S100A4*	BASE	*THAP2*	BASE
AIF1	BASE	*CD27*	BASE	*CXCL5*	BASE	*FOXP3*	BASE	*KRT6B*	BASE	*NKG7*	BASE	*S100A8*	BASE	*TIFA*	BASE
AKR1C1	BASE	*CD274*	BASE	*CXCR4*	BASE	*FSTL3*	BASE	*KRT7*	BASE	*NOSTRIN*	BASE	*SCD*	BASE	*TIGIT*	BASE
AKR1C3	BASE	*CD3D*	ADD-ON	*CYP1A1*	ADD-ON	*GATA3*	BASE	*KRT8*	BASE	*NPM3*	ADD-ON	*SCGB2A1*	BASE	*TIMP4*	BASE
ALDH1A3	BASE	*CD3E*	BASE	*CYTIP*	BASE	*GJB2*	BASE	*LAG3*	BASE	*OCIAD2*	BASE	*SDC4*	BASE	*TMEM147*	BASE
ANGPT2	BASE	*CD3G*	BASE	*DAPK3*	BASE	*GLIPR1*	BASE	*LARS*	BASE	*OPRPN*	BASE	*SEC11C*	BASE	*TNFRSF17*	BASE
ANKRD28	BASE	*CD4*	BASE	*DERL3*	ADD-ON	*GNLY*	BASE	*LDHB*	BASE	*OXTR*	BASE	*SEC24A*	BASE	*TOMM7*	BASE
ANKRD29	BASE	*CD68*	BASE	*DMKN*	BASE	*GPR183*	BASE	*LEP*	ADD-ON	*PCLAF*	BASE	*SELL*	BASE	*TOP2A*	BASE
ANKRD30A	BASE	*CD69*	BASE	*DNAAF1*	BASE	*GZMA*	BASE	*LGALSL*	BASE	*PCOLCE*	ADD-ON	*SERHL2*	BASE	*TPD52*	BASE
APOBEC3A	BASE	*CD79A*	BASE	*DNTTIP1*	BASE	*GZMB*	ADD-ON	*LIF*	ADD-ON	*PDCD1*	BASE	*SERPINA3*	BASE	*TPSAB1*	BASE
APOBEC3B	BASE	*CD79B*	BASE	*DPT*	BASE	*GZMK*	BASE	*LILRA4*	BASE	*PDCD1LG2*	BASE	*SERPINB9*	BASE	*TRAC*	BASE
APOC1	BASE	*CD80*	BASE	*DSC2*	BASE	*HAVCR2*	BASE	*LPL*	BASE	*PDE4A*	BASE	*SFRP1*	BASE	*TRAF4*	BASE
AQP1	BASE	*CD83*	BASE	*DSP*	BASE	*HDC*	ADD-ON	*LPXN*	BASE	*PDGFRA*	BASE	*SFRP4*	BASE	*TRAPPC3*	BASE
AQP3	BASE	*CD86*	BASE	*DST*	BASE	*HMGA1*	BASE	*LRRC15*	BASE	*PDGFRB*	BASE	*SH3YL1*	BASE	*TRIB1*	BASE
AR	BASE	*CD8A*	BASE	*DUSP2*	BASE	*HOOK2*	BASE	*LTB*	BASE	*PDK4*	BASE	*SLAMF1*	BASE	*TUBA4A*	BASE
AVPR1A	BASE	*CD8B*	ADD-ON	*DUSP5*	BASE	*HOXD8*	BASE	*LUM*	BASE	*PECAM1*	BASE	*SLAMF7*	BASE	*TUBB2B*	BASE
BACE2	BASE	*CD9*	BASE	*EDN1*	ADD-ON	*HOXD9*	BASE	*LY86*	BASE	*PELI1*	BASE	*SLC25A37*	BASE	*TYROBP*	ADD-ON
BANK1	BASE	*CD93*	BASE	*EDNRB*	ADD-ON	*HPX*	BASE	*LYPD3*	BASE	*PGR*	BASE	*SLC4A1*	ADD-ON	*UCP1*	BASE
BASP1	BASE	*CDC42EP1*	BASE	*EGFL7*	BASE	*IGF1*	BASE	*LYZ*	BASE	*PIGR*	BASE	*SLC5A6*	BASE	*USP53*	BASE
BTNL9	ADD-ON	*CDH1*	BASE	*EGFR*	BASE	*IGSF6*	BASE	*MAP3K8*	BASE	*PIM1*	BASE	*SMAP2*	BASE	*VOPP1*	BASE
C15orf48	BASE	*CEACAM6*	BASE	*EIF4EBP1*	BASE	*IL2RA*	BASE	*MDM2*	BASE	*PLD4*	BASE	*SMS*	BASE	*VWF*	BASE
C1QA	BASE	*CEACAM8*	ADD-ON	*ELF3*	ADD-ON	*IL2RG*	BASE	*MEDAG*	ADD-ON	*POLR2J3*	ADD-ON	*SNAI1*	BASE	*WARS*	BASE
C1QC	BASE	*CENPF*	BASE	*ELF5*	ADD-ON	*IL3RA*	BASE	*MKI67*	BASE	*POSTN*	BASE	*SOX17*	BASE	*ZEB1*	BASE
C2orf42	BASE	*CLCA2*	ADD-ON	*ENAH*	BASE	*IL7R*	BASE	*MLPH*	BASE	*PPARG*	BASE	*SOX18*	BASE	*ZEB2*	BASE
C5orf46	BASE	*CLDN4*	ADD-ON	*EPCAM*	BASE	*ITGAM*	BASE	*MMP1*	BASE	*PRDM1*	BASE	*SPIB*	BASE	*ZNF562*	BASE
C6orf132	BASE	*CLDN5*	ADD-ON	*ERBB2*	BASE	*ITGAX*	BASE	*MMP12*	BASE	*PRF1*	BASE	*SQLE*	BASE		
CAV1	BASE	*CLEC14A*	BASE	*ERN1*	BASE	*ITM2C*	BASE	*MMP2*	BASE	*PTGDS*	BASE	*SRPK1*	BASE		
CAVIN2	BASE	*CLEC9A*	BASE	*ESM1*	BASE	*JUP*	BASE	*MMRN2*	BASE	*PTN*	BASE	*SSTR2*	BASE		
CCDC6	BASE	*CLECL1*	BASE	*ESR1*	BASE	*KARS*	BASE	*MNDA*	BASE	*PTPRC*	BASE	*STC1*	BASE		
CCDC80	BASE	*CLIC6*	BASE	*FAM107B*	BASE	*KDR*	BASE	*MPO*	ADD-ON	*PTRHD1*	BASE	*SVIL*	BASE		
CCL20	ADD-ON	*CPA3*	BASE	*FAM49A*	BASE	*KIT*	BASE	*MRC1*	BASE	*QARS*	BASE	*TAC1*	BASE		
CCL5	BASE	*CRHBP*	ADD-ON	*FASN*	BASE	*KLF5*	BASE	*MS4A1*	BASE	*RAB30*	BASE	*TACSTD2*	BASE		

10x Development Teams

Consortium of 10x Genomics team members who all contributed to the development of technologies used in this manuscript.

Jawad Abousoud, Francis Aguisanda, Matt Alexander, Maria Alexis, Pratomo Alimsijah, Morris Allison, Aditya Aman, Joseph Aman, Zaid Ammari, Naishitha Anaparthy, Eric Anderson, Courtney Anderson, Melissa Ando, Joey Arthur, Adam Azarchs, Navi Bains, Naveed Bakh, Alexandru Bardasu, Carlos Barraza, Ashley Basco, David Batey, Florian Baumgartner, Alessio Bava, Kamila Belhocine, Jason Bell, Julio Beltran, Alex Ben, Zachary Bent, Casey (Jon) Berridge, Rajiv Bharadwaj, Anton Bjorninen, Kirk Blackmoore, Octavian Bloju, Lorita Boghospor, Jeff Bolland, Raj Borade, Erik Borgstrom, Sebastian Le Bras, Christian Broms, Phillip Brooks, Brenden Brown, Noel Harris Brown, Elizabeth Buckley, Patricia Buenbrazo, Ratha Bun, Diana Burkart-Waco, Jim Burrows, Michele Busby, Janae Bustos, Michele Caceres, Matthew Cai, Xiaoyang (Frank) Cai, Michael Campbell, Saranya Canchi, Joshua Cataldo, Emily Chan, Rena Chan, Alan Chang, Evelyn Chang, Ella Hsinwen Chang, Sharmila Chatterjee, Sidharth Chaturvedi, Mitu Chaudhary, James Chell, Kevin Chen, Sarah Chen, Hong Chen, Jui-Szu Chen, Sharon Chen, Brian Cheng, Jennifer Cheung, Jennifer Chew, Jun Ding Chiang, Hariharasudhan Chirra, Cheyenne Christopherson, Sonya Clark, Brynn Claypoole, Justin Costa, Julia Cowen, David Cox, Francis Cui, Abbey Cutchin, Aos Dabbagh, Smritee Dadhwal, Eileen Dalin, Aldo DeAmicis, Filip Defoort, Evan Dejarnette, Joshua Delaney, Nigel Delaney, Loruhama Delgado-Rivera, Maurizio Depoli, Abou Diop, Rhyan Dockter, Keri Dockter, Sultan Doganay, Jason Downing, Tyler Drake, Shoshoni Droz, Narek Dshkhunyan, Jens Durruthy Durruthy, Carine Edder, Peter Edge, Aaron Ellis, Emre Erhan, James Erhard, Cedric Espenel, Danielle Estes, Eric Evje, Navid Farahani, Reynaldo Farias, Dina Finan, Melanie Freeman, Danny Freitas, Tristan French, Meghan Frey, Alex Gagnon, Caroline Gallant, Abigail Gallegos, Christina Galonska, Karthik Ganapathy, Richard Gantt, Jason Gao, Kevin Gilmartin, Ryan Gilmore, Gabriele Girelli, Andreas Girgensohn, Michael Glazer, Shalini Gohil, Sumanth Gollapudi, Qiang Gong, Brandon Gordon, Erica Graziosa, Yang Gu, Josh Gu, Zhenping Guan, Zhengping Guan, Christopher Guido, Madhu Gundapuneni, Vaibhav Gupta, Rashmi Gupta, Lauren Gutgesell, Daniel Gyllborg, Lila Haba, Katherine Habeck, Andrew Haling, Katrín Halldórsdóttir, Andrej Hartnett, Ryo Hatori, Eileen He, Andrew Heiberg, Robert Henley, Janine Hensel, Lance Hepler, Alexander Hermes, Javier Hernandez, Iván Hernández, Jack Herrera, Jill Herschleb, Andrew Hill, Ben Hindson, Michael Hirte, Khoa Hoang, David Hoffman, Catie Hollyer, Eric Holt, Babak Honaryar, KC Hong, Daniel Horvath, Jennifer Hsu, Alice Huang, Winnie Hui, Kim Xuan Huynh, Hayato Ikoma, Moussa Iskandar, Eswar Iyer, Shaun Jackman, Sunil Jagannath, Samir Jain, Thomas Johnson, Tom Johnson, Guy Joseph, Aleksandra Jurek, Aarushi Kalaimani, Govinda Kamath, Lily Blanche Kameny, Ivar Karam, Rishabh Kasliwal, Layla Katiraee, Anna-Maria Katsori, Matthew Keeshen, Andrew Kessler, Tousif Khan, Aliyya Khan, Hanyoup Kim, Albert Kim, James Kim, Minji Kim, Sugyeom Kim, Alex Kindwall, Naga Sudha Kodavatikanti, Andrew Kohlway, Sutheng Kok, Sukhmal Kommidi, Nikola Kondov, Aishwarya Konnur, Jason Koth, Maria Kourbatov, Joseph Kovac, Sreenath Krishnan, Benjamin Ku, Jing Kuang, Malte Kuhnemund, Divya Anjan Kumar, Vijay Kumar, Josy Kuriakose, Sural Labha, Yves Lacroix, Karen Lai, Jana Lalakova, Tim Lam, Du Linh Lam, Alyssa Lanza, Jonathan Lau, Eunice Lau, Julia Lau, Lily Le, Soo Hee Lee, Thomas Lee, Josephine Lee, Junhyuck Lee,

Elizabeth Lee, Jennifer Lew, Meryl Lewis, Peigeng Li, Vera Li, Ziyang Li, Qiang Li, Yuwei Li, Dongyao Li, Alvin Liang, Varoth Lilascharoen, Marisa Lim, David Little, Kendra Liu, Evelyn Lo, Ludmila Lokteva, James Longmire, Glory Lopez, Steve Losh, Han Lu, Grace Lucas, Mike Lucero, Susana Lau Lui, Paul Lund, Yi Luo, Chris Macklin, Diego Magdaleno, Amanda Mah, Shamoni Maheshwari, Amit Maheshwari, Sarah Mahmoud, Michelle Mak, Liza Man, Arec Manoukian, Francis Marcogliese, Hendricus Marindra, Patrick Marks, Allison Martin, Kristen Martins-Taylor, Alicia McCarthy, Benjamin Mccreath, Shane McDermed, Jeff Mellen, Francesca Meschi, Paulius Mielinis, Marco Mignardi, Nabil Mikhaiel, Pradyumna Mishra, Adam Monkowski, Lysette Moreno, David Morgan, Logan Morrison, Fing Moua, Nima Mousavi, Aditi Mukherjee, Donald Mullane, Veronica Gonzalez Munoz, Laura Munteanu, Sivaram Muthusubramanian, Gambhir Nagaraju, Monica Nagendran, Jay Nagin, Sovann Nak, Akshay Nakra, Ashkan Beyranvand Nejad, Amber Nelson, Brenda Nguyen, Vu Nguyen, William Nitsch, Amy Oh, Jean-Francois Olivier, Mimmi Olofsson, Megan Olsen, Brett Olsen, James Ong, Susana Ordaz, Jessica Östlin, Dulce Ovando-Morales, Alex Palacio, Rushi Panchal, Janice Papartassee, Abhijna Parigi, Minkyung Park, Anuj Patel, Shyam Patel, Sid Patel, Kayne Patterson, Jeffrey Peck, Marissa Pennell, James Perna, Katherine Pfeiffer, Kristen Pham, Caio Porto, Denis Pristinski, Nicholas Provenzano, Benjamin Pruitt, Abhi Puthenveetil, Teddy De Puy, Xiaoyan Qian, Yufeng Qian, Ken Quah, Ailen Quero, Mohammad Rahimi, Selva Rajendran, Martino Ramella, Tina Ramirez, Jaya Ramrakhyani, Harjeet Randhawa, Nikhil Rao, Nicole Rapicavoli, Karl Rauta, Ann Renschler, Rudy Rico, Daniel Riordan, Elijah Roberts, Guillaume Robichaud, Anatalia Robles, Blas Rodrigues, Nancy Conejo Rodriguez, Mary Rogawski, Patrick Rolli, Michael Rose, Holly Ross, Chris Rouillard, Mustafa Rupawalla, Spontaneous Russell, Paul Ryvkin, Aprana Sahajan, Janani Sampathkumar, Marcus Sands, Poonam Sansanwal, Ace Santiago, Jerald Sapida, Anuj Sareen, Didem Sarikaya, Fahima Sarker, Hiroshi Sasaki, Martin Sauzade, Michael Schnall-Levin, Jason Schultz, Laurie Scott, Preyas Shah, Ankur Shah, Nathan Shapiro, Nate Shapiro, Shankar Shastry, Robert Shelansky, Kai Shen, Christian Shi, Shang Shi, Lisa Shi, Shreyas Shivalkar, Steve Short, Joe Shuga, Anton Shutov, Jordan T. Sicherman, Sana Siddiqi, Eric Siegel, Aisling Sinclair, Hardeep Singh, Pal Singh, Ben Sisserman, Alex Skrynnyk, Carl Skuce, Peter Smibert, Adam Smiechowski, Benjamin Smyth, Kelvin Soong, Delia Soto, Mario de Souza, Rapolas Spalinskas, Susanne Spiegelberg, Nico Sponer, Niranjan Srinivas, Yogesh Srinivas, Jasper Staab, William Stanislaus, Jacob Stern, Marlon Stoeckius, Ryan Stott, Zachary Strike, Arjun Sugumar, David Sukovich, Ilse Sweldens, Kokchuan Tan, Qiaoqiao Tan, Sarah Tang, Weiyi (Lily) Tang, Adrian Tanner, Raghu Tayanna, Sarah Taylor, Ryan Taylor, Nolan Teasdale-Schaf, Augusto Tentori, Jessica Terry, Joshua Thao, Michael Tierney, Meiliana Tjandra, Mckenzi Toh, Jeremy Tong, Joaquin Torres, Thien Trac, Khoi Tran, Christopher Tran, Joaquin Trosper-Torres, Andriy Tsupryk, Hank Tu, Mesruh Turkekul, Cedric Uytingco, Dino Valdecanas, Miriam Valencia, Ben Veire, Toon Verheyen, Rajiv Verma, Jan Vigar, Priya Rajendran Vishnu, Olga Vorobyova, Florian, Wagner, Dagmar Walter, Amywenli Wang, Su Wang, Jun Wang, Dylan Webster, Jason Weis, Neil Weisenfeld, Kevin West, Tobias Wheeler, Dieter Wilk, Stephen R. Williams, Chris Wing, Christopher Wing, Evan Winget, George Withers, Tiffany Wong, Alex Wong, Philip Wright, Snow Wu, Kevin Wu, Sherry Wu, Fen Xie, Xiankun Xu, Shawn Yackly, RaviTeja Yarlagadda, Jennifer Yeager, Yifeng Yin, Tingsheng Yu Drennon, Frances Yun, Brett Zaborsky, Negin Zaraee, Ryan Zeng, Meng Zhang, Thanutra Zhang, Yiran Zhang, Fuying Zheng, Orchid Zhu, and Yihui Zhu.

Supplementary Reference List

1. Pal, B. *et al.* A single-cell RNA expression atlas of normal, preneoplastic and tumorigenic states in the human breast. *EMBO J.* **40**, e107333 (2021).
2. Bhat-Nakshatri, P. *et al.* A single-cell atlas of the healthy breast tissues reveals clinically relevant clusters of breast epithelial cells. *Cell Rep. Med.* **2**, 100219 (2021).
3. Karlsson, M. *et al.* A single–cell type transcriptomics map of human tissues. *Sci. Adv.* **7**, eabh2169 (2021).
4. Cable, D. M. *et al.* Robust decomposition of cell type mixtures in spatial transcriptomics. *Nat. Biotechnol.* **40**, 517–526 (2022).
